# Clinical outcomes and prognostic significance of early *vs.* late computed tomography in acute pancreatitis

**DOI:** 10.1093/gastro/gou067

**Published:** 2014-10-10

**Authors:** Vishal Sharma, Surinder S. Rana, Ravi K. Sharma, Rajesh Gupta, Deepak K. Bhasin

**Affiliations:** ^1^Department of Gastroenterology, Postgraduate Institute of Medical Education and Research, Chandigarh 160012, India and ^2^Department of Surgery, Postgraduate Institute of Medical Education and Research, Chandigarh 160012, India

**Keywords:** acute pancreatitis, computed tomography, complications, clinical outcomes

## Abstract

**Background:** Guidelines recommend that contrast-enhanced computed tomography (CT) should be carried out 72 hours after onset of an attack of acute pancreatitis (AP). However, the exact time beyond 72 hours at which CT will produce the best diagnostic yield for local complications, or whether doing a CT early in acute pancreatitis has any adverse effect on the course of disease, is not clear.

**Methods:** The medical records of 214 consecutive patients with AP were analysed retrospectively and these patients were divided into two groups: the early CT group (CT done at 4–5 days after the onset of pain) and the late CT group (CT done in days 6–14 following onset of pain). The two groups were compared for differences in clinical outcomes and prognostic information obtained from CT, such as detection of pancreatic necrosis and local complications, and CT severity index.

**Results:** Of 214 patients [143 (66.8%) males; mean age 39.87 ± 13.52 years], 21 patients were excluded as they did not undergo CT or CT was done more than 14 days after onset of an attack of AP. The early CT group included 114 patients, whilst the late CT group had 79. The mean CT severity index was higher in the late CT group (6.65 ± 2.27 *vs.* 5.52 ± 2.7; *P = *0.005). The incidence of persistent organ failure in the early group was no different from that of the late group (38.6% *vs.* 49.4%; *P = *0.143). Local complications were detected more often in the late CT group (84.8% *vs.* 68.4%; *P = *0.011). There was no difference between the two groups in the need for percutaneous drainage, surgery, or mortality (all *P > *0.05).

**Conclusions:** Although performing early CT does not adversely affect the outcome in AP, CT carried out more than 5 days after the onset of symptoms may detect more local complications.

## Introduction

Acute pancreatitis (AP) is an important gastroenterological emergency which, if severe, can lead on to a variety of local and systemic complications, a prolonged hospital course, and significant morbidity and mortality [1]. Pancreatic necrosis and peripancreatic fluid collections are the important local complications of AP and patients with pancreatic necrosis have markedly increased morbidity and mortality when compared with patients with non-necrotizing interstitial pancreatitis [2, 3]. The pancreatic necrosis is currently best detected by the loss of vascular enhancement when using cross-sectional imaging such as computed tomography (CT) or magnetic resonance imaging [1]. CT using intravenous contrast also provides an opportunity to diagnose additional pancreatic complications such as splanchnic thrombosis, extrapancreatic necrosis, ascites, pleural effusion, fluid collection and, later, walled-off necrosis/pseudocysts and pseudoaneurysms [4, 5].

However, on cross-sectional imaging, the signs of pancreatic necrosis evolve over several days, and their full extent may therefore not be completely apparent on a contrast-enhanced CT for up to 3 days after the onset of disease. Most guidelines therefore recommend that CT be done more than 72 hours after onset of symptoms. However, the development of pancreatic necrosis and local complications in AP is a dynamic process and the exact time beyond 72 hours following onset of symptoms—at which CT will show the best diagnostic yield for local complications—is not clear [1, 3, 4].

Animal studies have also shown that the intravenous contrast medium sometimes used in CT aggravates the severity of the disease by impairing pancreatic microcirculation, and these studies suggested that contrast-enhanced CT should not be performed in the early phase of acute pancreatitis [6, 7]. Experimental and clinical studies subsequently performed have both refuted and supported this claim of the detrimental effect of early CT in acute pancreatitis and this issue is still under debate [8, 9]. However, recent studies have shown that early CT does not aggravate acute pancreatitis and, on the contrary, provides important prognostic information [10, 11]. We conducted this retrospective study to compare clinical outcomes, as well as prognostic information, obtained by performing early CT (within 5 days of the onset of pain) with late CT (done 6–14 days after onset of pain).

## Patients and methods

A retrospective analysis was carried out, of the collected data on the patients with AP at our unit from July 2012 to December 2013. The diagnosis of AP was established on the basis of the presence of any two of three findings (typical abdominal pain, amylase/lipase elevation at least three times normal, or consistent radiological findings) [1]. We excluded patients who did not undergo abdominal computed tomography (e.g. because of renal failure or pregnancy), patients undergoing CT more than 14 days after onset of pain or having underlying chronic pancreatitis/pancreatic malignancy. The study protocol was approved by our institute's Ethics Committee.

The patients presented to us at variable times after the onset of an attack of AP and it follows that contrast-enhanced CT was carried out at correspondingly variable time points. Patients presenting within 72 hours of onset underwent CT 3 days after the attack, whereas patients presenting later underwent CT on the day of admission. The severity of disease was staged according to the Balthazar Computed Tomography Severity Index (CTSI) [4].

The demographic, clinical, laboratory and radiological data for the included patients were collected, as were details of the treatment received. The outcome was noted vis-à-vis organ failure, local complications, need for radiological/endoscopic intervention, surgery and mortality. The patients were divided into two groups: early CT group (CT done within 5 days of onset of pain), and late CT group (between days 6–14 after onset of pain). The two groups were compared for various outcome parameters, such as organ failure, local complications, need for radiological/endoscopic intervention, surgery and mortality.

## Statistical analysis

The descriptive data was presented as percentages for categorical variables, and mean ± standard deviation (SD) for quantitative variables. The continuous variables were compared using student's *t*-test, whereas the categorical variables were compared using the Chi-squared test. A *P*-value of < 0.05 was considered as significant.

## Results

Two hundred and fourteen consecutive patients [143 males (66.8%); mean age 39.87 ± 13.52 years) with AP were seen during the study period. The aetiology implicated in the causation of acute pancreatitis was alcohol in 88 patients (41.1%), gall stones in 62 (29.0%), both alcohol and gall stones in 14 (6.5%), idiopathic in 22 (10.3%) and other origin in 28 (13.1%). At admission, 177 (82.7%) showed evidence of systemic inflammatory response syndrome (SIRS) and the mean bedside index for severity in acute pancreatitis (BISAP) score was 2.11 ± 0.90. Of these 214 patients, 21 were excluded (CT not done in 9 patients and CT done more than 14 days after onset of attack of AP in 12) and 193 patients were included for final analysis. These were divided into two groups: the early CT group included 114 patients (77 males; mean age: 40.36 ± 14.34 years) whilst there were 79 patients in the late CT group (52 males; mean age: 40.01 ± 12.12 years). The mean timeframes in which the CT was carried out in the early and late CT groups were 3.66 ± 1.12 days and 11.78 ± 2.47 days, respectively.

Demographic profiles and baseline parameters were comparable between these two groups (Table 1). There were no significant differences between the early and late CT groups in terms of age, underlying aetiology, haematological (haematocrit; leukocyte count) or biochemical parameters (blood urea; serum creatinine). The differences in clinical presentation (pain; fever and jaundice) were also not significant between the two groups, except for the frequency of the presence of mass, which was higher in the early CT group (34.2% *vs.* 58.2%; *P = *0.001). As for parameters predicting severe acute pancreatitis, the two groups had similar SIRS (80.7% and 82.2%; *P = *0.852) and mean BISAP (2.11 ± 0.94 *vs.* 2.09 ± 0.92; *P = *0.903) at presentation. However, the mean CTSI was significantly lower in the early CT group (5.52 ± 2.7 *vs.* 6.65 ± 2.27; *P = *0.005).

**Table 1.** Comparison of demographic profile and baseline parameters between the two groups

	Early CT (*n* = 114)	Late CT (*n* = 79)	*P*-value
Age (years)	40.36 ± 14.34	40.01 ± 12.12	0.863
Male, *n* (%)	77 (67.5)	52 (65.8)	0.877
Aetiology, *n* (%)			0.095
Alcohol	43 (37.7)	35 (44.3)	
Gall Stones	33 (28.9)	23 (29.1)	
Haematocrit (%)	35.76 ± 8.28	33.56 ± 7.35	0.059
Total leukocyte count (per mm^3^)	15858 ± 6640	15981 ± 7826	0.906
Serum albumin (g/dL)	3.46 ± 1.69	3.15 ± 0.55	0.120
Blood urea (mg/dL)	41.7 ± 33.9	47.2 ± 55.6	0.396
Serum creatinine (mg/dL)	1.16 ± 1.66	1.24 ± 1.59	0.738
Pain, *n* (%)	113 (99.1)	79 (100)	1.00
Mass, *n* (%)	39 (34.2)	46 (58.2)	0.001
Jaundice, *n* (%)	7 (6.1)	2 (2.5)	0.313
SIRS, *n* (%)	92 (80.7)	65 (82.2)	0.852
Pleural effusion, *n* (%)	98 (85.9)	70 (88.6)	0.667
Ascites, *n* (%)	66 (57.9)	53 (67.1)	0.175
BISAP	2.11 ± 0.94	2.09 ± 0.92	0.903
CTSI	5.52 ± 2.7	6.65 ± 2.27	0.005

SIRS = systemic inflammatory response syndrome; BISAP = bedside index for severity in acute pancreatitis; CTSI = computed tomography severity index

These patients were subsequently followed up and the outcome parameters compared between the two groups (Table 2). The frequency of persistent organ failure in the early CT group was no different from the late group (38.6% *vs.* 49.4%; *P = *0.143). Local complications (acute fluid collections, as well as acute necrotic collections) were detected more often in the late group (84.8% *vs.* 68.4%; *P = *0.011) than in the early CT group (84.8% *vs.* 68.4%; *P = *0.011). The need for percutaneous drainage (20.2% *vs.* 30.3%; *P = *0.125) or the need for surgery (7.0% *vs.*12.6%; *P = *0.125) was similar in both groups. Also, there was no statistically significant difference in mortality rates between the two groups (13.1% *vs.*10.1%; *P = *0.653).

**Table 2:** Comparison of outcome parameters between the two groups

	Early CT (*n* = 114)	Late CT (*n* = 79)	*P*-value
Local complications, *n* (%)	78 (68.4)	67 (84.8)	0.011
Persistent organ failure, *n* (%)	44 (38.6)	39 (49.4)	0.143
Percutaneous drainage, *n* (%)	23 (20.2)	24 (30.3)	0.125
Surgery, *n* (%)	8 (7.0)	10 (12.6)	0.213
Mortality, *n* (%)	15 (13.1)	8 (10.1)	0.653

## Discussion

Most guidelines recommend that contrast-enhanced CT of the abdomen be done more than 72 hours after onset of symptoms as CT carried out earlier may underestimate the extent of pancreatic necrosis. However, the development of pancreatic necrosis and local complications in AP is a dynamic process and the exact time beyond 72 hours after onset of symptoms, at which CT will show the best diagnostic yield for local complications, is not clear [1, 3, 4]. The studies have advocated a widely varying interval from the onset of acute attack to performing CT scan, ranging from 72 hours to 10 days [12, 13]. The best time to perform CT of the abdomen in AP—so that the best results are obtained for evaluating the severity of acute pancreatitis—therefore remains uncertain.

In the present study, we found that CT performed more than 5 days following onset of symptoms diagnosed more local complications (acute fluid-, as well as necrotic collections, than CT done after day 3 following onset of symptoms). Also the mean CTSI was significantly higher in the late CT group than in the early group. The CTSI comprises two components: first the Balthazar grade, which also takes into account the presence of peripancreatic collections; second, the necrosis score and this difference in CTSI could be because of increased frequency of detection of acute fluid collections, as well as of necrotic collections, in the late CT group. The CTSI, a 10-point scoring system, has been shown to be an important prognostic indicator in AP [14, 15]. Although it is generally agreed that the development and extent of pancreatic necrosis are the most important indicators of disease severity in AP, one study has shown that 22% of patients with CT grades of D or E developed complications despite the absence of pancreatic necrosis [14]. Therefore, for accurate prognostication, it is important to determine both the local complications and the extent of pancreatic necrosis. Pancreatic necrosis usually develops 2–4 days after the onset of an acute attack and rarely progresses. Therefore, CT performed after 3 days would be able to delineate pancreatic necrosis accurately, but we have shown that local complications can be best detected by CT performed 5 days after the onset of an AP attack [14]. In our centre, therefore, we now do not carry out CT at admission, but 5 days after the onset of AP.

There has also been speculation that injection of contrast may result in worsening of pancreatic necrosis by affecting pancreatic microcirculation: initial animal studies have indeed demonstrated that intravenous contrast medium given during CT aggravated the severity of the disease. Experimental and clinical studies performed subsequently have both refuted as well as supported this claim of detrimental effect of early CT in acute pancreatitis and this issue is still under debate [8–11].

In a report on a rat model of cerulean-induced pancreatitis, the injection of contrast seemed to increase the rates of pancreatic necrosis and mortality [6]. However in a taurocholate-induced model of experimental pancreatitis, the authors have suggested that neither ionic nor non-ionic contrasts increase pancreatic necrosis [16]. Recent clinical studies have shown that early CT does not aggravate AP and, on the contrary, provides important prognostic information [10, 11]. We also observed that early CT did not aggravate the course of AP and the outcome parameters vis-à-vis the need for percutaneous intervention, surgery and mortality were no different in the two groups. Although our study has a fairly large sample size of 193 patients but retrospective analysis and a non-randomised design are important limitations of the current study.

In conclusion, although performing early CT does not adversely affect the outcome in AP, CT should be done 5 days after an attack as the frequency of detection of local complications of AP is higher than with CT done within 5 days of onset of symptoms.

*Conflict of interest statement:* none declared.
